# Editorial: Human Disorders of PI3K Biology

**DOI:** 10.3389/fimmu.2020.617464

**Published:** 2020-11-26

**Authors:** Carrie L. Lucas, Stuart G. Tangye

**Affiliations:** ^1^Department of Immunobiology, Yale University School of Medicine, New Haven, CT, United States; ^2^Immunity & Inflammation Theme, Garvan Institute of Medical Research, Darlinghurst, NSW, Australia

**Keywords:** PI3K, primary immunodeficiencies, Activated PI3Kδ Syndrome, immunology, human genetics

The aim of this Research Topic was to bring together experts in basic, translational, and clinical research relating to phosphoinositide 3-kinase (PI3K) biology. The monogenic human immune disease “Activated PI3Kδ Syndrome” (APDS) has shed new light on immune functions of this kinase as well as its therapeutic targeting. PI3Ks have pleotropic effects across all cell types by phosphorylating PtdIns(4,5)P_2_ to generate PtdIns(3,4,5)P_3_, a second messenger that recruits and activates signaling proteins to trigger cell growth, proliferation, and survival. PI3Kδ is comprised of the p110δ catalytic and the p85α regulatory subunits, encoded by *PIK3CD* and *PIK3R1*, respectively. This kinase complex can be targeted by multiple small molecule inhibitors, many of which are currently at various stages of clinical development. Heterozygous variants in *PIK3CD* or *PIK3R1* have been found to cause APDS, primarily by affecting inhibitory contacts between the two proteins. Defining the genetic etiology of this disorder has enabled rational targeted therapy to tune down PI3Kδ signaling.

These and related discoveries have not only advanced our understanding of human disease but also pointed to gaps in basic science knowledge regarding regulation of PI3K complexes and their activity. In this Research Topic, 13 manuscripts cover a range of subjects, mostly centered around findings in APDS and cancer. Michalovich and Nejentsev discuss genetic discovery as a basis for treatment of APDS, which includes hematopoietic stem cell transplantation (HSCT), management of infections/lymphoproliferation, and targeted inhibition of PI3Kδ in clinical trials. Dornan and Burke review structural biology concepts for Class IA PI3K variants in cancer and immunodeficiency. Various biochemical and biophysical studies have shown the intricate molecular mechanisms by which class IA PI3Ks are regulated via intra- and inter-subunit interactions between the catalytic and regulatory subunits. The differential expression of PI3Ks, in addition to their varied response to upstream activating stimuli, contributes to their regulation. Thus, further elucidation of these mechanisms is crucial as PI3Ks are linked to various human diseases ranging from developmental disorders, to cancer and immunodeficiencies.

Condliffe and Chandra review respiratory manifestations of APDS. The vast majority of APDS patients present with early-onset recurrent respiratory infections of bacterial and viral origin due to compromised immune responses, leading to complications such as bronchiectasis and small airway disease. Malignant or benign lymphoproliferative disease, along with other non-infectious conditions such as growth impairment, are also common in APDS patients. Maccari et al. provide a perspective piece on the European Society for Immunodeficiencies (ESID) APDS registry and highlight disease evolution and response to rapamycin. The chronology of presentation for these conditions usually begins with recurrent respiratory infections very early in life, followed by lymphoproliferative disorders, and then with gastrointestinal conditions and autoimmune cytopenias. Inhibition of mechanistic target of rapamycin (mTOR), a regulator of cell proliferation and growth downstream of PI3Kδ, with rapamycin (sirolimus) is effective at mitigating lymphoproliferative disease in APDS but has had limited effect on managing other features of disease. Coulter and Cant discuss in more depth potential therapeutic approaches. APDS patients have various clinical manifestations with some patients being asymptomatic while others exhibiting recurrent infections and antibody defects. Historically, conventional therapies such as immunoglobulin replacement therapy, HSCT, and antimicrobial prophylaxis have been used as treatments. However, the heterogeneity of disease presentation requires a more tailored approach which can be achieved through the use of selective PI3Kδ inhibitors such as Leniolisib.

Wentink et al. provide new data on the phenotype of CD8^+^ T cells in APDS as it relates to exhaustion. As a contributing mechanism for increased susceptibility to infections and dysregulated immune responses, CD8^+^ T cell exhaustion due to chronic T cell stimulation and proliferation is relevant for APDS pathology. Cannons et al. provide a perspective piece on the survival, differentiation, and function of CD8^+^ T cells in APDS. Despite having a normal or even elevated frequency of Epstein-Barr Virus (EBV)–specific CD8^+^ T cells, APDS patients have defects in controlling EBV and cytomegalovirus viremia. While seemingly not affecting the development of antigen-specific T cells, hyperactive PI3Kδ impacts CD8^+^ T cell proliferation, differentiation, and survival, which have direct relevance for their function *in vivo*. Cohen covers consequences of herpesvirus infections in APDS in more depth. Herpesviruses can directly bind surface receptors that activate the PI3Kδ pathway and further modulate signaling through viral proteins. Together with compromised antibody production, cytokine secretion, and phagocytosis in APDS, these effects may contribute to the prevalence of uncontrolled herpesviruses in this disorder.

Mace addresses natural killer (NK) cells in the context of PI3K signaling and its role in the migration, activation, signaling and cytotoxicity of NK cells. Dysregulation of these pathways has important impacts on viral infections and malignancy. Fan and Turka discuss PI3K in regulatory T cells. In addition to the effects of APDS on the function of B cells, macrophages, and various other T cell compartments, recent studies have begun to elucidate the nuanced relationship between metabolic pathways and the function and lineage maintenance of Tregs as directed by IL-2 signaling through effects of PI3Kδ on FOXP3 expression. This highlights the possibility of targeting particular subsets of T cells based on their preferred metabolic pathways, potentially allowing for either strengthening or dampening Treg suppressive activities to combat autoimmune conditions or boost immune activation, respectively.

Asano et al. provide new data on APDS B cells and hyperactive PI3Kδ in this key cell type. The level of phosphorylated AKT (pAKT) is reported to be higher in unstimulated circulating B cells of patients with APDS compared to healthy controls, as assessed by phospho-flow cytometry. This may allow for the differentiation between the various forms of APDS resulting from different pathogenic variants. Since this assay does not require culturing patient cells, it opens the possibility for using the level of pAKT as a rapid diagnostic tool. Jhamnani et al. discuss class switch recombination (CSR) phenotypes in APDS. Since APDS patients exhibit defects in CSR with elevated IgM and low IgG, IgA, and IgE, it can be categorized within the spectrum of hyper-IgM syndromes.

Jung et al. review a set of immune diseases affecting function of mTOR. They discuss variants in genes encoding components of the PI3K/AKT/mTOR/S6 kinase (S6K) signaling pathway(s) that have been associated with primary immunodeficiencies. The overlapping immunodeficiency phenotypes observed in patients with impairment in these pathways led to the suggested disease category of “immune TOR-pathies.”

Together, these articles address findings related to genetics, structural biology, clinical manifestations and treatments, CD8 T cell responses, NK cell biology, T regulatory cells, B cell abnormalities, and multiple precision treatment perspectives.

## Author Contributions

CLL and SGT wrote and edited the editorial. All authors contributed to the article and approved the submitted version.

## Funding

CLL was funded by Yale University and NIAID/NIH grants R01AI138141 and R21AI144315. SGT is supported by grants awarded by the National Health and Medical Research Council of Australia.

## Conflict of Interest

The authors declare that the research was conducted in the absence of any commercial or financial relationships that could be construed as a potential conflict of interest.

